# Bacterial microbiomes of *Ixodes scapularis* ticks collected from Massachusetts and Texas, USA

**DOI:** 10.1186/s12866-019-1514-7

**Published:** 2019-06-24

**Authors:** Santosh Thapa, Yan Zhang, Michael S. Allen

**Affiliations:** 1 Tick Borne Disease Research Laboratory, Department of Microbiology, Immunology and Genetics, University of North Texas Health Science Center, Fort Worth, TX USA; 2 Present Address: Texas Children’s Microbiome Center, Department of Pathology, Texas Children’s Hospital, Houston, TX USA; 3 Present Address: Department of Pathology and Immunology, Baylor College of Medicine, Houston, TX USA

**Keywords:** Tick microbiome, *Ixodes scapularis*, 16S rRNA, Microbial diversity, Blacklegged tick, Ecology

## Abstract

**Background:**

The blacklegged tick, *Ixodes scapularis*, is the primary vector of the Lyme disease spirochete *Borrelia burgdorferi* in North America. Though the tick is found across the eastern United States, Lyme disease is endemic to the northeast and upper midwest and rare or absent in the southern portion of the vector’s range. In an effort to better understand the tick microbiome from diverse geographic and climatic regions, we analysed the bacterial community of 115 *I. scapularis* adults collected from vegetation in Texas and Massachusetts, representing extreme ends of the vector’s range, by massively parallel sequencing of the 16S V4 rRNA gene. In addition, 7 female *I. scapularis* collected from dogs in Texas were included in the study.

**Results:**

Male *I. scapularis* ticks had a more diverse bacterial microbiome in comparison to the female ticks. *Rickettsia* spp. dominated the microbiomes of field-collected female *I. scapularis* from both regions, as well as half of the males from Texas. In addition, the male and female ticks captured from Massachusetts contained high proportions of the pathogens *Anaplasma* and *Borrelia*, as well as the arthropod endosymbiont *Wolbachia*. None of these were found in libraries generated from ticks collected in Texas. *Pseudomonas*, *Acinetobacter* and *Mycobacterium* were significantly differently abundant (*p <* 0.05) between the male ticks from Massachusetts and Texas. *Anaplasma* and *Borrelia* were found in 15 and 63% of the 62 Massachusetts ticks, respectively, with a co-infection rate of 11%. Female ticks collected from Texas dogs were particularly diverse, and contained several genera including *Rickettsia*, *Pseudomonas*, *Bradyrhizobium*, *Sediminibacterium*, and *Ralstonia*.

**Conclusions:**

Our results indicate that the bacterial microbiomes of *I. scapularis* ticks vary by sex and geography, with significantly more diversity in male microbiomes compared to females. We found that sex plays a larger role than geography in shaping the composition/diversity of the *I. scapularis* microbiome, but that geography affects what additional taxa are represented (beyond *Rickettsia*) and whether pathogens are found. Furthermore, recent feeding may have a role in shaping the tick microbiome, as evident from a more complex bacterial community in female ticks from dogs compared to the wild-caught questing females. These findings may provide further insight into the differences in the ability of the ticks to acquire, maintain and transmit pathogens. Future studies on possible causes and consequences of these differences will shed additional light on tick microbiome biology and vector competence.

**Electronic supplementary material:**

The online version of this article (10.1186/s12866-019-1514-7) contains supplementary material, which is available to authorized users.

## Background

Ticks transmit a greater variety of human and animal pathogens than any other disease vector and have significant public health impacts worldwide [1–7]. The blacklegged tick, *Ixodes scapularis*, is the major vector of the Lyme disease agent, *Borrelia burgdorferi*, in North America [8]. This tick also carries a number of other pathogens that cause human disease, including agents of anaplasmosis, babesiosis and Powassan encephalitis [9–16].

With more than 30,000 reported cases per year and an estimated 10-fold greater burden than the reported case counts, Lyme disease is the most common vector-borne illness in the U.S. [1, 17–19]. Despite a broad geographic distribution of *I. scapularis* across the eastern United States, Lyme disease cases are concentrated in the northeastern and upper midwestern states, whereas the disease is very rare or absent in the southern portion of the vector’s range [9, 19]. The prevalence of *B. burgdorferi* among *I. scapularis* in the northeastern U.S. has been reported to be as high as 30–50% [20–22], while it is rarely (< 1%) detected in the ticks from the southern United States [23–25]. Over the past two decades, the incidence of Lyme disease has increased in numbers and geographical area across the eastern U.S., which coincides with a significant northward range expansion of *I. scapularis* in the northeastern and midwestern regions [9, 26].

Many factors, including the density of host-seeking *B. burgdorferi-*harboring ticks, availability of the *B. burgdorferi* competent hosts, tick-behavior, seasonal activity of the ticks, and environmental variables influence the risk of Lyme disease [9, 27–33], yet the reasons behind the regional distribution of Lyme disease are not fully understood. In the upper midwestern and northeastern U.S., all active stages of *I. scapularis* can be encountered by humans during warm season of the year. But, in the southeastern U.S., human encounter occurs primarily with the adults *I. scapularis* ticks as immature ticks rarely seeks hosts in the region [34]. Recent studies have demonstrated that the resident microbial community of ixodid ticks can influence reproductive fitness and physiological processes of the tick and the acquisition, establishment and transmission of certain tick-borne pathogens [4, 35–39]. The microbial community of *I. scapularis* ticks has increasingly been studied in recent years [40–43]. In the U.S., the microbiome of *Ixodes* ticks varies with sex, species and geography [44]. By contrast, in Canada the microbiomes of *I. scapularis* ticks from eastern and southern Ontario do not differ significantly with regard to geographic origin, sex or life stages [40]. These contradicting reports highlight the need for additional studies considering the potential role that geography and related ecological and environmental factors may have in shaping the microbiome of ixodid ticks and disease transmission. More recently, we have demonstrated that the composition of the endogenous tick microbial community in colony-reared *I. scapularis* can be influenced by the environmental temperature [45]. With that goal in mind, we investigated the bacterial microbiomes of *I. scapularis* adults collected from natural vegetation from Texas and Massachusetts, representing opposing ends of the vector’s range and possessing distinct climates, by sequencing the hypervariable region 4 (V4) of the 16S ribosomal RNA (rRNA) gene using an Illumina MiSeq platform. Adult ticks were chosen to provide a fair comparison of the tick microbiomes from two regions with different geography and climate, and to provide ample DNA per sample without the need for pooling of multiple, smaller life stages.

Host blood meal has been shown to affect microbial diversity in *I. pacificus* [39], a closely related species to *I. scapularis*, with potential consequences for vector competence. To investigate how host blood meal affects microbiome, we also analysed the bacterial microbiomes of dog-fed female *I. scapularis* ticks during this study.

## Results

### 16S V4 sequencing results

From the 122 *I. scapularis* samples (115 questing *I. scapularis* adult ticks collected from Texas and Massachusetts plus 7 female ticks collected from dogs in Texas, see Table 2 in Methods section for details) 12,204,399 quality-filtered reads (average per sample = 100,036; standard deviation = 24,411; range = 29,611- 167,874) were generated. This library included 6544 reads generated from negative controls (one blank extraction control and another no-template PCR negative control). The number of reads (for a particular OTU) that were present in the negative controls was subtracted from the libraries of the samples. Additionally, for genus level data analysis, 0.085% of the reads from each sample were considered as zero to minimize putative background contamination (i.e. if an OTU was abundant at less than 0.085% in a given sample, it was removed from the downstream analyses). All libraries generated from tick samples had adequate depth for further analysis, as evident from the mean Good’s coverage of 99.9% (range = 99.9–100%). Additionally, rarefaction curves of the number of observed OTUs plotted at a depth from 1000 to 30,000 sequences reaching plateau ~ 25,000 reads (Additional file 1: Figure S1) suggested sufficient sample coverage to proceed further.

### Bacterial composition of *I. scapularis*

Proteobacteria dominated the *I. scapularis* microbiomes in both locations under study. Proteobacteria were prevalent at 87.2% mean relative abundance across field-collected ticks from Texas, with 73% (3.5–96.5%) prevalence in males and 100% (99.9–100%) prevalence in females. The prevalence of Proteobacteria in female ticks collected from dogs in Texas was 93% (86–100%). Proteobacteria were prevalent at 84.8% across ticks from Massachusetts, with 71.9% (0–99.9%) prevalence in males and 98.5% (11.1–88.9%) in females. Other common phyla in the microbiomes of the Texas ticks (both field-collected males and the females from dogs) included various proportions of Actinobacteria, Bacteroidetes, and Firmicutes. In addition to these non-proteobacterial phyla found in the Texas ticks, Massachusetts ticks were represented by the Spirochaetes, albeit with higher abundance in males (63% of them with ≥1% relative abundance) compared to that of the females (37%) (see Additional file 1: Figure S2).

**Fig. 1:**
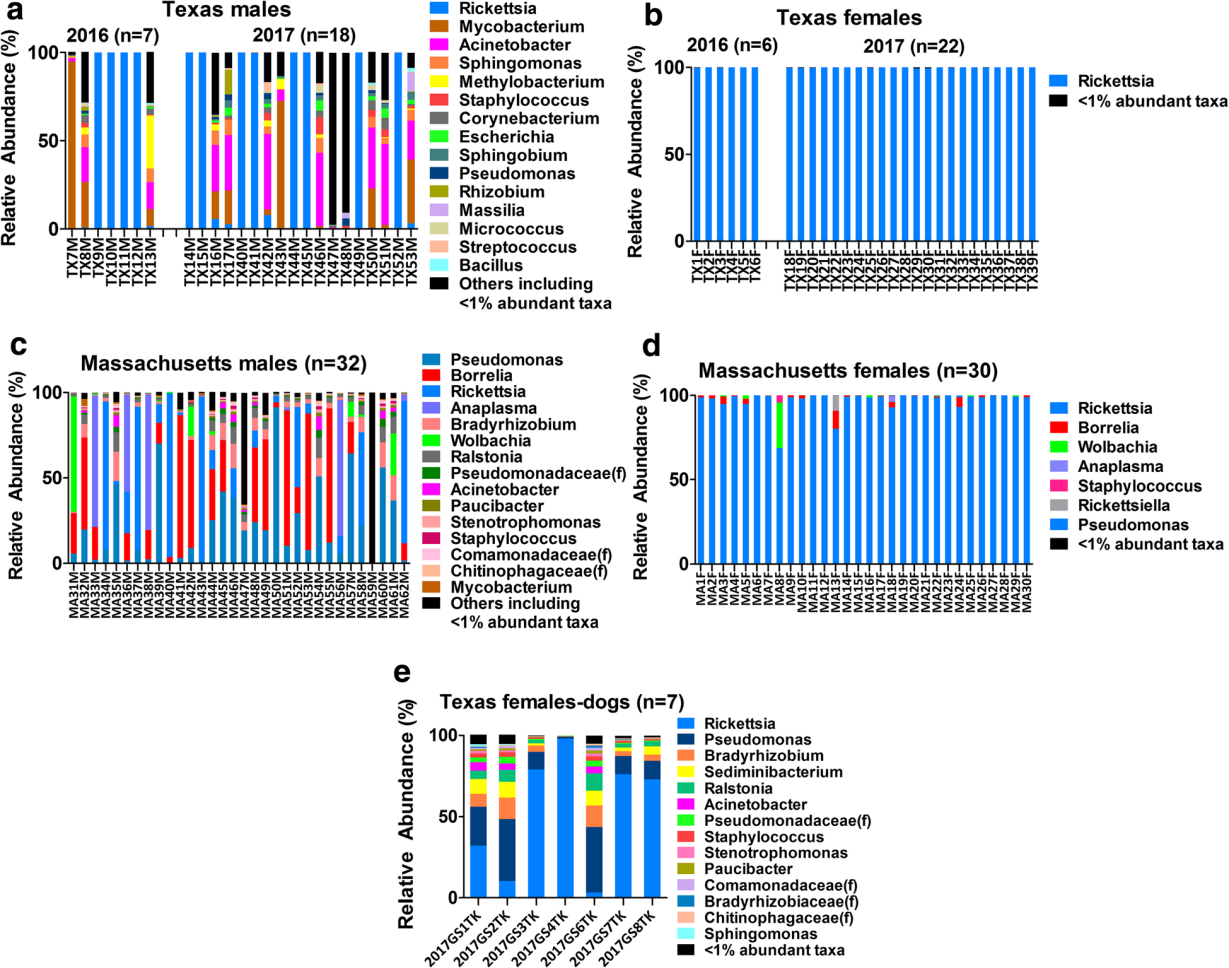
Genus-level relative abundance of bacteria in *I. scapularis* ticks collected from Texas and Massachusetts. Field-collected male (**a**) and female (**b**) ticks from TX are compared with the males (**c**) and females (**d**) from MA, as well as the females collected from dogs (**e**) in TX. Each bar represents an individual whole tick (identified on the x-axis) where different colors indicate the percentage of 16S V4 rRNA gene sequences assigned to each genus (whenever possible) based on 97% identity to taxa in the Greengenes reference database. Only the top-15 dominant taxa are shown for the male ticks, while all taxa ≥1% relative abundance are shown for the females. (f = family unclassified)

In field-collected Texas ticks, *Rickettsia* spp. almost entirely dominated the microbiome of all the female ticks (Fig. 1). Although 66 taxa, including 48 genera, had an abundance of ≥1% in at least one of the 25 male samples from Texas, about half of them were also entirely dominated by *Rickettsia*. The remaining Texas male ticks (*n* = 13) had a more complex microbiome comprised of *Mycobacterium* (mean = 23%), *Acinetobacter* (22%), *Sphingomonas* (5%)*, Methylobacterium* (4%), *Corynebacterium* (2%), *Staphylococcus* (2%), *Escherichia* (2%), *Rickettsia* (2%), *Sphingobium* (2%), *Rhizobium* (1%), *Pseudomonas* (1%), and others. There was the lack of variation in microbiome composition of field-collected Texas ticks between the collection years (Kruskal-Wallis test *p* > 0.05 for both male and female ticks (see Fig. 1) thus justified lumping ticks of the same sex but different years into the same category.

Libraries generated from ticks collected in woodlands in Massachusetts showed a similar dependence on the sex of the host. *Rickettsia* dominated the female microbiome (mean relative abundance = 97%, range = 69–100%), followed by *Borrelia* (overall mean < 2%), *Wolbachia* (1%), *Anaplasma* (0.1%), and others (Fig. 1). While *Rickettsia* was present in all female ticks from Massachusetts, *Borrelia* and *Anaplasma* were detected in 60 and 10% of the female samples, respectively. The mean relative abundance of *Borrelia* and *Anaplasma* among the positive female samples was 2% (0.2–1%) and 1% (2–79%), respectively. *Wolbachia*, with a mean relative abundance of 1.1% (0–27%), was found in more than 25% of the females from the region. Microbiomes of male ticks collected in Massachusetts were represented by multiple genera, including *Pseudomonas* (overall mean relative abundance = 23%), *Borrelia* (23%), *Rickettsia* (20%)*, Anaplasma* (9%), *Bradyrhizobium* (5%) *Wolbachia* (4%), *Ralstonia* (4%), *Acinetobacter* (1%), and others. Altogether, 17 genera and 7 additional unclassified taxa (4 unclassified families, one unclassified at class-level and two unclassified at the phylum-level) had 1% or greater abundance in at least one of the 32 libraries generated from male ticks collected in Massachusetts. *Borrelia* (mean relative abundance among positive samples = 35%, range = 0.1–84%) was found in 66% of the Massachusetts males, while *Anaplasma* (50%, 2–80%) was detected in about 19% of the males. Moreover, *Wolbachia* was also found in about 28% of the male ticks from Massachusetts.

Significant differences were found in the mean relative abundance of certain genera in males from Texas and Massachusetts including, *Pseudomonas* (Kruskal-Wallis test *p =* 0.0001), *Acinetobacter* (*p =* 0.006) and *Mycobacterium* (*p =* 0.004). Additionally, *Anaplasma*, *Borrelia* and *Wolbachia* bacteria were found in both male and female ticks from MA, but not from those in TX.

Libraries generated from female ticks collected from dogs in Texas contained many genera that were rare or absent in libraries generated from female ticks collected from foliage in Texas and Massachusetts. These actively feeding ticks did carry *Rickettsia* (mean abundance = 53%), but the abundances of *Pseudomonas* (20%), *Bradyrhizobium* (7%), *Sediminibacterium* (5%), *Ralstonia* (4%), and *Acinetobacter* (2%) were much higher than in the microbiomes of questing ticks collected from foliage (Fig. 1).

### Bacterial composition in *I. scapularis* females after removal of *Rickettsia*

**Fig. 2:**
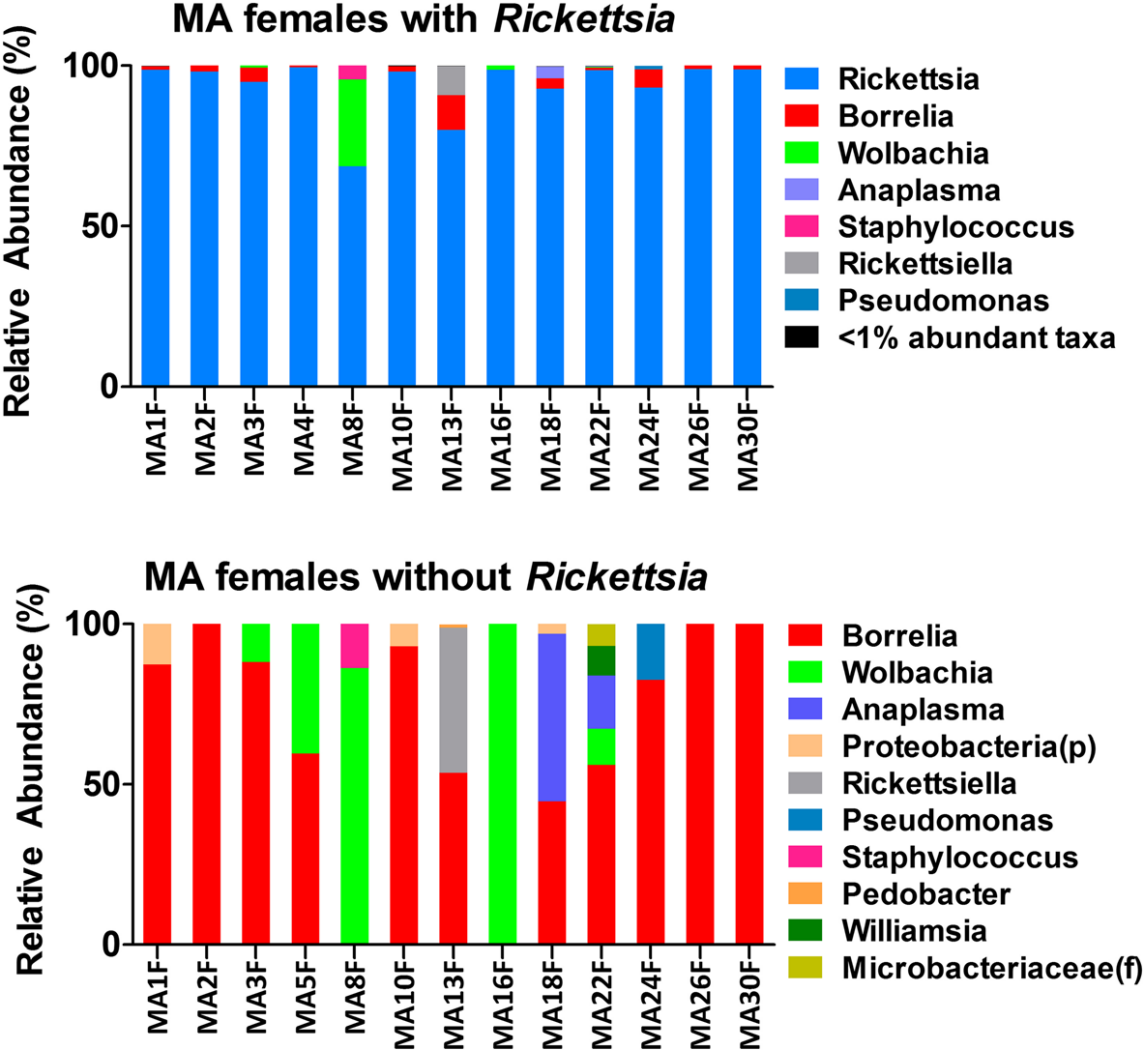
Composition of bacterial taxa identified in field-collected female *I. scapularis* ticks from Massachusetts with *Rickettsia* (upper panel) and after *in-silico* removal of *Rickettsia* (lower panel). Taxa are classified to the genus level whenever possible and each bar represents an individual tick. Only ticks with at least 1000 non-rickettsial sequences are shown. See text for details

Because the microbiomes of female ticks were entirely dominated by amplicons likely derived from the rickettsial endosymbiont known to occur in this species, and because this endosymbiont resides primarily in the ovaries [46], we removed *Rickettsia* sequences from the female data sets to further explore the inherent (predominately) gut microbiome of the female ticks, as described in Thapa et al. (2018) [45]. After *in-silico* removal of *Rickettsia* sequences from the female dataset*,* none of the field-collected female samples from Texas had sufficient sequences remaining to pass the inclusion criteria (as described in the beginning of the results section) needed to proceed for further analysis. Five female samples from Massachusetts also did not meet the inclusion criteria for further analysis after removing *Rickettsia*. Of the remaining 25 female samples, all collected from Massachusetts, only 13 had more than 1000 reads (mean = 7439, range = 1149–29,487) after deleting *Rickettsia* sequences. *In-silico* removal of *Rickettsia* from the Massachusetts female datasets revealed the previously hidden bacterial composition (Fig. 2), where the presence of *Borrelia* was prominent compared to the full female profiles (i.e. *Rickettsia* included). In contrast to a very low distribution of *Borrelia* in their full profiles (range = 0.5–11%, mean = 3%), the relative abundance of *Borrelia* in 11 *Rickettsia*-deleted female samples (two of the 13 samples under analysis were negative for these bacteria) ranged from 45 to 100% (mean = 79%) (Fig. 2).

### Alpha diversity

**Fig. 3:**
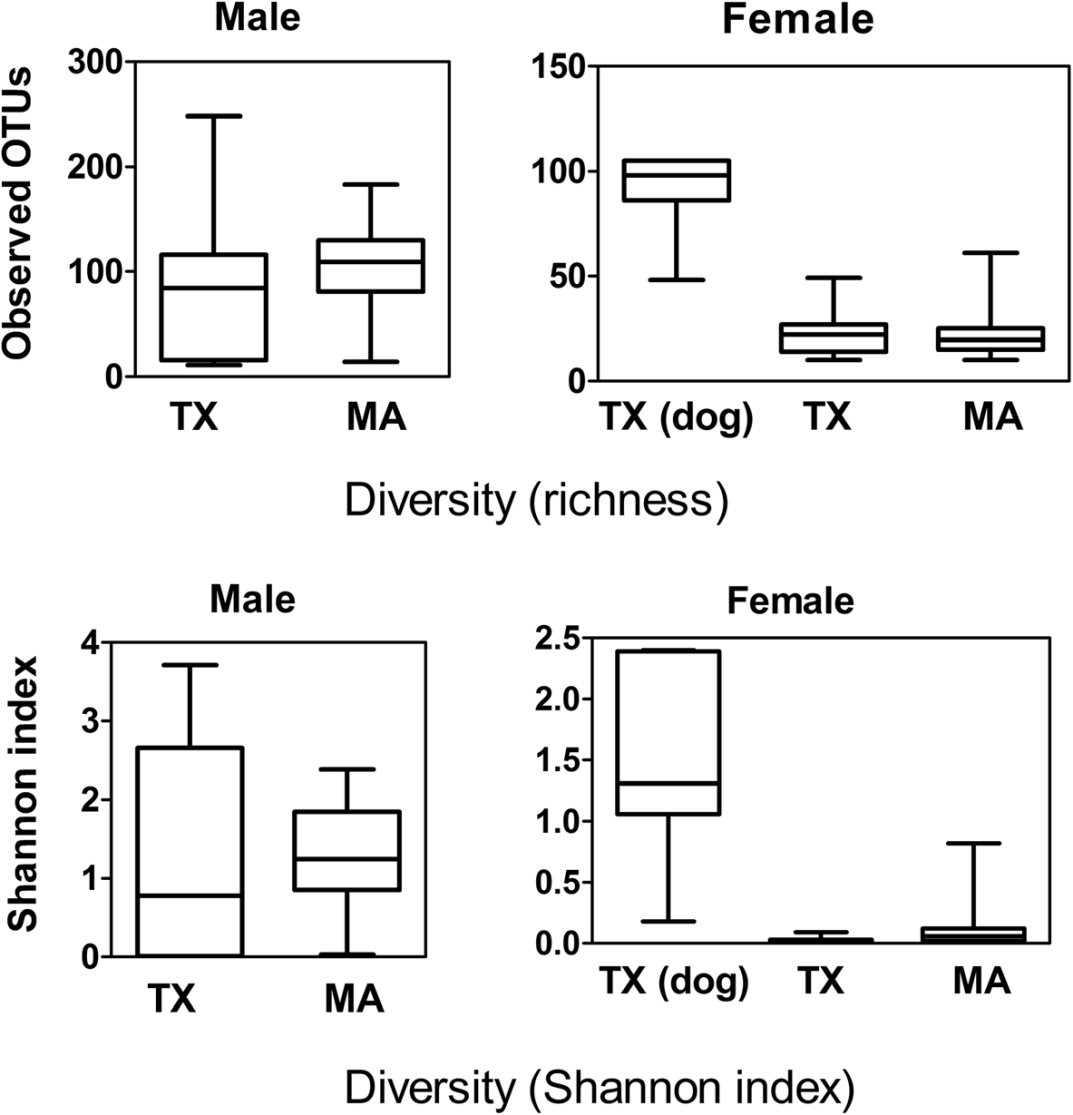
Alpha diversity measures for male and female ticks

Prior to diversity analyses, subsampling at the minimum sequencing depth (25,059 sequences per sample) was performed to normalize the number of sequences in each sample [47]. Regardless of their geographical origin, field-collected male ticks exhibited significantly higher bacterial richness (number of OTUs observed) than that of the females (Wilcoxon rank-sum test *p <* 0.0001). However, female ticks collected from dogs in Texas had a significantly higher number of observed OTUs in comparison to the foliage-associated female ticks from either Texas or Massachusetts (FDR corrected Wilcoxon rank-sum test *p <* 0.0001 for all comparisons) (see Fig. 3). Similar results were found with ACE (abundance-based coverage estimator) value and Chao1 estimator (See Additional file 1: Figure S3). The Shannon diversity index of male ticks from both sites was significantly higher than the females (Wilcoxon rank-sum test *p <* 0.0001 for both sites compared separately) (Fig. 3), and female ticks collected from foliage in both Texas and Massachusetts had a lower bacterial diversity in comparison to the female ticks collected from dogs in Texas. Further multiple comparison analyses revealed no significant differences in the bacterial diversity of female ticks collected from dogs with that of the male ticks collected from foliage in both states.

### Beta diversity

**Fig. 4:**
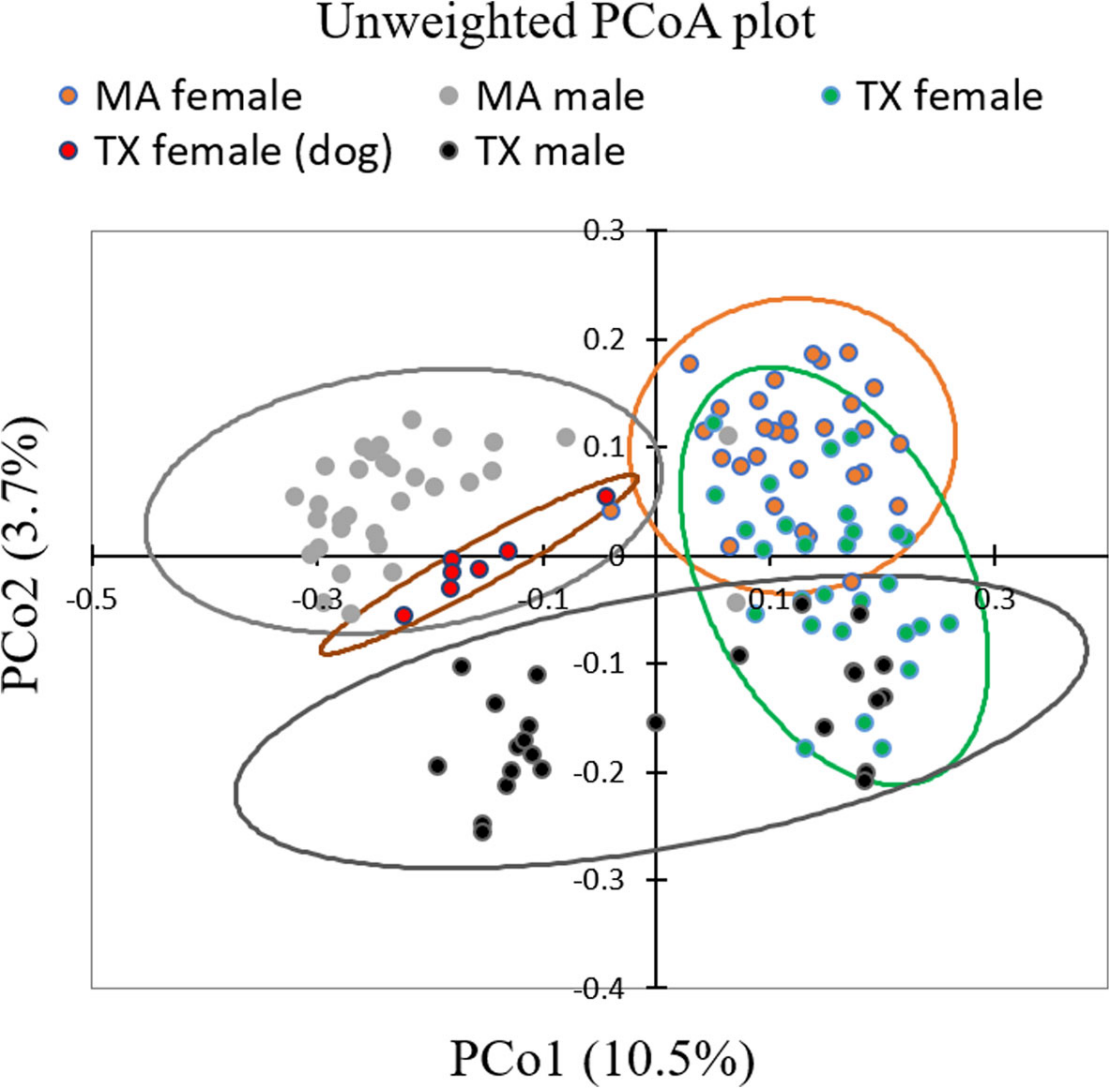
PCoA plot using unweighted UniFrac distance of the bacterial composition in male and female ticks. Texas males are clustered separately from the Massachusetts male ticks; Female ticks collected from dogs in Texas (TX female (dog)) also clustered separately from the field-collected female ticks from both Texas and Massachusetts. Ellipses indicate 95% confidence intervals. Each dot represents the bacterial microbiome of an individual tick

While cluster analyses did not show a clear separation of the ticks for all samples, the majority of the male *I. scapularis* ticks collected from Massachusetts clustered separately from others, as did half the males from Texas in an unweighted PCoA plot of axis-1 vs axis-2. PCoA of unweighted UniFrac distances of bacterial communities showed that the first two axes (PCo1 and PCo2) explained 10.5 and 3.7% of the variation in the data, respectively (Fig. 4). PERMANOVA analysis of Unweighted UniFrac distances revealed a significant difference in the microbiome composition of male and female ticks from both collection sites (Adonis *p* = 0.001). The plot also demonstrated that the male samples from TX clustered separately when compared to the males from Massachusetts (*p* = 0.001), except one outlier from MA within the cluster of TX males. Female ticks collected from dogs in Texas formed a coherent cluster close to the field-captured males from Massachusetts. No clear clustering was observed in a PCoA plot of the weighted UniFrac distance metrics based on the collection site or sex of the tick (See Additional file 1: Figure S4).

### Co-infection of *Borrelia* and *Anaplasma* in Massachusetts ticks

*Anaplasma* and *Borrelia* were detected exclusively in libraries generated from ticks collected in Massachusetts. *Anaplasma-Borrelia* appeared together in 7 of 62 (11%) field-collected *I. scapularis* from Massachusetts (Table 1).

**Table 1:** Distribution of *Borrelia* and *Anaplasma* in field-collected *I. scapularis* ticks from Massachusetts

Sex	No. of *I. scapularis*	*Borrelia* +ve	*Anaplasma* +ve	*Borrelia-* *Anaplasma* + ve
Male	32	21 (66%)	6 (19%)	5 (16%)
Female	30	18 (60%)	3 (10%)	2 (7%)
Total	62	39 (63%)	9 (15%)	7 (11%)

### Microbiome of *Borrelia*-positive and *Borrelia*-negative *I. scapularis* ticks

Although the distribution of individual bacterial taxa varied among male and female ticks from Massachusetts, there was no significant difference in bacterial composition between *Borrelia*-positive and *Borrelia*-negative groups in both male (PERMANOVA of UniFrac distance, Adonis *p =* 0.25) and female (*p =* 0.26). (Additional file 1 Figure S5). However, *Borrelia*-negative males from Massachusetts had a different community structure of bacteria when compared to the Texas males based on PERMANOVA test of the unweighted UniFrac distance metrics (Adonis *p* = 0.001). In terms of female ticks, *Rickettsia* was almost exclusively dominant in both regions, but Massachusetts females contained *Borrelia*, *Anaplasma,* and *Wolbachia*, while these bacteria were absent from the ticks collected in Texas.

### Microbiome comparisons of the colony-reared and field-collected *I. scapularis* ticks

We also compared the baseline microbiome data of the colony-reared *I. scapularis* ticks from our previous publication [45] to that of the microbiome data obtained from the field-collected ticks in the present study. In the case of the male ticks, relative abundance of Bacteroidetes and Firmicutes was significantly different in colony-reared ticks than that of the wild-caught ticks from Texas or Massachusetts (BH corrected Dunn’s Kruskal Wallis test *p* < 0.05 for all comparisons). In female ticks, Proteobacteria dominated the microbiome of both colony-reared and field-collected ticks (questing and dog fed) (See Additional file 1: Figure S6). Similar to the Texas ticks, *Borrelia*, *Anaplasma,* and *Wolbachia* were not found in the colony-reared ticks purchased from the Tick Rearing Facility at Oklahoma State University (OSU). Bacterial diversity in colony-reared male ticks found to differ significantly from that of the wild-caught males from TX or MA (unweighted UniFrac PERMANOVA *p* = 0.001 for both comparisons). The colony-reared female ticks also differed significantly in beta diversity (as measured by unweighted UniFrac distances) when compared to that of the female ticks collected from vegetation in TX and MA (*p* = 0.001). However, the colony-reared *I. scapularis* females were no more diverse than the female ticks collected from dogs (*p* = 0.06).

## Discussion

The dominance of Proteobacteria in *I. scapularis* ticks from both Texas and Massachusetts agrees with a previous study [44] of wild-caught ticks from several U.S. states that also found > 80% of the reads could be assigned to Proteobacteria. Other phyla found in this study, including Spirochaetes, were also previously reported in wild-caught ticks [44]. Our finding of exclusive abundance (100%) of *Rickettsia* in all field-collected female ticks from Texas, and a very high dominance (97%) in females from Massachusetts, is consistent with previous reports for the microbiome of *I. scapularis* [41–44] and high prevalence of *Rickettsia* in larvae and nymphal *I. scapularis* [6, 22].

The high numbers of *Rickettsia* likely reflect a mutualism between this endosymbiont and the host, and most likely belong to the endosymbiont *Rickettsia buchneri* [46]. *R. buchneri* has been shown to provide a source of vitamins to the tick [48]. The genus *Rickettsia* also contains many potentially pathogenic species, including *Rickettsia rickettsii, R. japonica, R. akari* [49] and *R. parkeri* [50]*,* but these bacteria are not known to be vectored by *I. scapularis*. By contrast, high prevalence of *R. buchneri* endosymbionts in female ticks is generally associated with the ovaries [41, 51]. As *Rickettsia* was also highly prevalent in male ticks, our findings suggest that *Rickettsia resides* in other body parts of the male ticks. This is consistent with previous reports of *R. parkeri* detected in male tissues of *Amblyomma maculatum* [52].

The complexity of the microbiomes of male ticks collected in both Massachusetts and Texas may reflect acquisition from the environment, as relatives of many genera found in the guts of male ticks are considered free-living (not host-associated) bacteria. Differences, such as the relative abundance of *Pseudomonas* (MA = 23% vs TX = 1%), *Acinetobacter* (MA = 1% vs TX =22%), *Mycobacterium* (MA = 0.4% vs TX = 23%) and the exclusivity of *Borrelia* and *Anaplasma* to MA, suggests a geographical and/or ecological variation of the microbiota in these ticks with public health consequences. Our findings of slightly higher abundance of *Borrelia* or *Anaplasma* in male ticks from Massachusetts compared to the female ticks suggest the possible roles of the underlying microbial community in the male ticks for pathogen acquisition. However, it should also be noted that some differences between males and females may be artifacts arising from differences in sequencing depth between males and rickettsia-subtracted females. Caution is therefore warranted in interpretation of these differences. In addition, the large variation between microbiomes of wild-caught Texas male ticks suggests the possibility of two distinct microbiomes. However, all the ticks were collected from the same habitat in Texas during 2016 and 2017, and the pattern is similar for both collection years. The variations between the microbiomes of Texas male ticks might have to do with prior host blood meal. The mean abundance of *Borrelia*, which could include pathogenic *B. burgdorferi* and *B. miyamotoi¸* was higher in males (35%) in comparison to the females (< 2%) and in the range of previous studies [20, 41, 44]. Xu et al. (20) study tested the ticks via qPCR while our study is based on 16S sequencing. So, the methodological differences could also have contributed to the relatively high levels of *Borrelia* and *Anaplasma* detected in *I. scapularis* ticks collected from Massachusetts. In comparison to the traditional PCR-based approaches used previously [20], the 16S rRNA gene sequencing used here cannot discriminate between species. It is highly likely that the samples that yielded *Borrelia* 16S rRNA gene sequences are due to *B. burgdorferi* (the causative agent of Lyme disease), but could be partially due to *B. miyamotoi* (relapsing fever group bacterium). *B. miyamotoi* has also been identified in this area, albeit at substantially lower numbers, with 2.3% of ticks tested from Cape Cod in 2016 found positive for this bacterium [53]. Furthermore, not all *I. scapularis* samples yielding *Borrelia* reads from 16S rRNA Illumina sequencing produce amplicons in PCR testing of the *B. burgdorferi* specific *ospC* gene [44]. Similar results of discordance between traditional PCR assays and Illumina MiSeq sequencing was also observed in another study on the *A. americanum* tick [54]. However, our findings of about 63% *Borrelia* in *I. scapularis* ticks collected from North Truro in Cape Cod, Massachusetts is in line with the findings of Xu et al. (2016) [20], who also reported that 62.5% of *I. scapularis* ticks tested from Nantucket county in Massachusetts were *B. burgdorferi* positive, and also consistent with unpublished work conducted by our laboratory using nested PCR methods (data not shown).

The *Anaplasma-Borrelia* co-infection rate of 11% in the ticks from Massachusetts we report was substantially higher than a previous study [20] on human-biting *I. scapularis* from Massachusetts, where 1.8% of the ticks were coinfected by *B. burgdorferi* and *A. phagocytophilum*. The higher rate of co-infection in this study could be attributed to the overall higher prevalence of *Borrelia*.

The detection of *Wolbachia* in more than 25% of the ticks from Massachusetts was not expected. *Wolbachia* are known to exhibit endosymbiotic mutualism with insects [55, 56], and has been previously reported in other ticks [57, 58], but not *I. scapularis*. Although *Wolbachia* has been known to induce resistance to dengue virus when introduced into *Aedes aegypti* mosquitoes [56] and other insects [55], Plantard et al. (2012) showed that *Wolbachia* in the *I. ricinus* tick, a major European vector of the Lyme disease agent, is due to the presence of the endoparasitoid wasp *Ixodiphagus hookeri,* and not representative of a true endosymbiont of the tick [59]. Thus, the prevalence of *Wolbachia* reported here is likely not a true mutualism with *I. scapularis*, but rather may indicate the presence of an unidentified parasite.

The higher bacterial richness in the microbiome of male ticks compared to female ticks, regardless of the geographical origin reflects dominance of *Rickettsia* in female ticks. Furthermore, a significantly higher Shannon diversity in male ticks suggests that the community of male ticks were more diverse as well as even, compared to the females.

The complexity of libraries generated from female *I. scapularis* ticks collected from dogs in Texas, in comparison to the wild-caught females from both states, suggests that the tick microbiome may shift as the result of a recent blood meal. The microbiomes of the female ticks that originated from dogs in Texas closely matches that of the male ticks from Massachusetts in terms of diversity but not in community membership, further supporting the idea that the bacterial microbiomes of female *I. scapularis* ticks vary with their sample source. One possible explanation could be that recent blood feeding led to increased abundance of midgut bacteria, lessening the overall relative impact of the rickettsial endosymbiont on subsequent analyses.

Difference in the composition and diversity of the microbiome of colony-reared *I. scapularis* ticks in comparison to the wild-caught ticks could be attributed to multiple factors, including the type of previous blood meal, and the environmental/ecological parameters. The difference in microbiomes of *I. scapularis* ticks from Massachusetts and Texas, including the ticks fed on dogs in Texas may, also reflect the seasonal effects on the tick microbiome. Indeed, we have previously shown that the environmental temperature can influence the endogenous tick microbial community composition in colony-reared *I. scapularis* [45].

## Conclusions

Analyses of the microbiomes of field-collected adult *I. scapularis* ticks from Texas and Massachusetts demonstrated that the bacterial microbiota of the ticks varies by sex and geographic origin. The main findings of this study are that sex plays a larger role than geography in shaping the composition/diversity of the *I. scapularis* microbiome, but that geography affects what additional taxa are represented (beyond *Rickettsia*) and whether pathogens are found. In addition, the microbiome of dog-fed female *I. scapularis* ticks is more complex than those of the wild-caught females.

Taken together, our findings may provide further insight into the sexual and regional differences in the ability of the ticks to acquire, maintain and transmit pathogens. Future studies on functional and mechanistic aspects of the tick microbiome, including possible causes (such as the ecological factors) and consequences of these differences will help us better understand the microbiome biology of the ticks and vector competence. These efforts may ultimately aid development of strategies to control the risk and transmission of tick-borne diseases.

## Methods

### Tick sampling and processing

During 2016 and 2017, a total of 115 adult *I. scapularis* were collected in the Davy Crockett National Forest near Kennard, Texas and from the North Truro area in the Barnstable county of Cape Cod, Massachusetts. Due to the difference in activity levels of ticks in different local environments, sample collection in Massachusetts was done during the late spring while ticks from Texas were collected during autumn. *I. scapularis* is endemic to both Cape Cod, located in the northeast U.S. [20, 60], and Trinity county in Texas, part of the southeastern U.S. [9]. Standard flagging technique was used for tick sampling, which consisted of walking down trails dragging a 1 m^2^ piece of white cloth attached to a pole gently over and around the vegetation where ticks were likely to be present. All encountered ticks were collected with fine-tipped tweezers and placed into sterile collection vials containing cotton fabric for housing. Ticks were categorized by location (TX or MA) and sex (male or female). All ticks were then preserved at ˗20 °C until DNA extraction. In addition, seven *I. scapularis* females collected from dogs (pulled off with tweezers) in North Texas were included in the study. These dog-fed ticks were unengorged to partially engorged. Details of the collection sites and dates are provided in Table 2

**Table 2:** *Ixodes scapularis* ticks collected from different sites in Texas and Massachusetts, USA

Collection sites	County	Source	Collection year	No. of ticks by sex	Total no. of ticks
Davy Crockett	Trinity	Vegetation	December 2017	males (14), females (11)	
			November 2017	males (4), females (11)	53
			December 2016	males (7), females (6)	
Hurst, Texas	Tarrant	Dogs	December 2017	Females (7)	7
North Truro in Cape Cod, Massachusetts	Barnstable	Vegetation	May 2017	Males (32), females (30)	62

.

### DNA extraction

All tick samples were treated in sequence with 10% sodium hypochlorite and molecular biology grade water to reduce surface contamination. Sterilization techniques using sodium hypochlorite solution have previously been demonstrated to significantly eliminate the bacteria and DNA on the tick surface [61]. Each whole tick was then cut into sections with a sterile scalpel on a glass microscope slide to disintegrate the thick cuticle layer and all sections were used during DNA extraction as previously described [45]. Briefly, all resultant sections of a tick were placed in a 2-ml screw-capped FastPrep tube (MP Biomedicals, LLC., Santa Ana, CA) containing 550 μl CSPL® buffer (Omega Bio-tek, Norcross, GA) and 8–10 sterile 2.8 mm ceramic beads (MoBio Laboratories Inc., Carlsbad, CA). Following pulverization (3 cycles of 7 m/s for 60s) in a FastPrep-24™ 5G Instrument (MP Biomedicals, LLC.), each sample was incubated at 56 °C for 2 h. Total DNA was then extracted from 122 individual ticks using a Mag-Bind® Plant DNA Plus Kit (Omega Bio-tek) as per the manufacturer’s instructions. A blank extraction control with reagents and beads was also prepared for each lot of DNA extractions. The extracted genomic DNA was quantified with a Nanodrop spectrophotometer (Invitrogen, Carlsbad, CA) and stored at − 20 °C until further processing.

### Tick mitochondrial 16S rRNA gene amplification

Each DNA extract was first assessed by PCR to amplify the tick mitochondrial 16S rRNA gene as a sample positive control, as previously described [45] using 16S-1 and 16S + 2 primers [62].

### Bacterial 16S rRNA gene amplification

DNA was amplified in duplicates by PCR using 515F/806R primers that target the hypervariable region four (V4) of the bacterial 16S rRNA gene. The primer set (forward: 5′-GTGCCAGCMGCCGCGGTAA-3′ and reverse 5′-GGACTACHVGGGTWTCTAAT-3′) had overhanging Illumina sequencing adaptors. Earth Microbiome Project (EMP) 16S Illumina Amplification Protocol was followed [63] with minor modifications as described below. In brief, a master mix solution was prepared per 25-μl PCR reaction volume with 2.5-μl 10X Accuprime™ PCR Buffer II (Invitrogen, Carlsbad, CA), 2.5-μl of 1.6 mg/ml Bovine Serum Albumin (New England Biolabs, Inc., Ipswich, MA), 1-μl 50 mM MgSO_4_, 0.5-μl 10 μM forward primer, 0.5-μl 10 μM reverse primer, 0.1-μl of 5 U/μl Accuprime™ *Taq* DNA Polymerase High Fidelity, 10-μl (43–554 ng) of template DNA and 7.9-μl molecular biology grade water. PCR was carried out in a BioRad C1000 Touch™ thermal cycler with the following cycling parameters: an initial denaturation at 94 °C for 2 min followed by 30 cycles (35 cycles for all male samples, with few exceptions, 40 cycles) consisting of denaturation at 94 °C for 30 s, annealing at 55 °C for 40 s, and extension at 68 °C for 40 s, with a final extension at 68 °C for 5 min and a 4 °C indefinite hold. Amplicon quality was evaluated by visualizing under UV light after separation in 1.5% agarose gel after electrophoresis. No template negative controls were used during the PCR runs.

### 16S rRNA gene library preparation and sequencing

PCR amplicons in duplicate sets were combined for each sample. Purification of the PCR products were performed using AMPure XP magnetic beads, and 16S libraries for a total of 122 samples were prepared following the Illumina 16S metagenomic sequencing library preparation protocol with the following minor modifications: the reaction mixture for index PCR (per 50 μl reaction) consisted of 5-μl 10X Accuprime™ PCR Buffer II, 5-μl Nextera XT Index Primer 1, 5-μl Nextera XT Index Primer 2, 0.2-μl Accuprime™ *Taq* DNA Polymerase High Fidelity (5 U/μl), 5-μl PCR product and 29.8-μl molecular biology grade water. Amplification was performed in a BioRad C1000 Touch™ thermocycler with the following program: 94 °C for 3 min followed by 8 cycles of 94 °C for 30 s, 55 °C for 30 s, 68 °C for 30 s, and 68 °C for 5 min, followed by holding at 4 °C. The final purified PCR products were quantified using a Qubit® 2.0 fluorometer (Thermo Fisher Scientific Inc., Carlsbad, CA) and libraries were pooled in equimolar amounts. The pooled library (10 pM final concentration) was loaded in the MiSeq Reagent Kit v2 (Illumina Inc., San Diego, CA) and paired-end (2 × 250) sequencing (500 cycles) was performed on an Illumina MiSeq instrument per manufacturer’s recommendations. Two negative controls (a blank extraction control and a no-template PCR negative control) were sequenced alongside of the samples to monitor for background contamination. A 5% PhiX DNA was also included in the run as an internal control.

### Data analysis

Data were analyzed in the Windows version of Microsoft Excel 2016 (Microsoft Corporation, Redmond, WA), XLSTAT-Ecology (Addinsoft SARL, NY), and R software (version 3.4.1) as well as in mothur software [64]. GraphPad Prism (v5.04) was also used to generate visuals. Raw sequences generated out of the Illumina MiSeq Instrument were processed using mothur v1.36.1. Quality filtered merged reads were aligned to the SILVA database (Release 132) [65] and chimeras were removed using the UCHIME [66] algorithm. Sequences within a 97% identity threshold were binned into operational taxonomic units (OTUs) [67] and taxonomic groups were assigned by comparison to the Greengenes reference database v13.8.99 [68, 69].

*Rickettsia* sequences were removed from the dataset using the remove.lineage command in mothur, as described in Thapa et al. (2018) [45]. Relative abundances of bacterial taxa were then compared between groups based on location (Texas vs Massachusetts), sex (male vs female), and source (vegetation vs dogs). Taxa with < 1% relative abundance in all samples were grouped together into ‘< 1% abundant taxa’ category for visual representation. Alpha diversity within samples was calculated using Observed OTUs, ACE value, Chao1 estimator, and Shannon index [70] in the data set rarefied at the lowest sequencing depth of 25, 059 reads/sample. Beta-diversity beween samples was quantified by weighted and unweighted UniFrac distance matrices and the bacterial community structure was visualized using principal coordinates analysis (PCoA) plots. Statistical analyses of the differently abundant taxa among groups were performed using the Kruskal Wallis test. Comparison between groups was performed using the Wilcoxon rank-sum test. Permutational multivariate analysis of variance (PERMANOVA) was used to determine the differences in microbial community composition within and among the groups using the ‘Vegan’ (v2.5.3) and ‘PhyloSeq’ (v1.24.2) R-packages. If appropriate, a post-hoc correction using the Benjamini-Hochberg method [71], which takes into account the false discovery rate (FDR) [72], was applied for multiple comparison testing [73]. The level of significance used in these analyses was 0.05.

## Additional file

Additional file 1: **Figure S1.** Rarefaction curves of the number of OTUs observed in male and female *I. scapularis.* **Figure S2.** Relative abundance of bacterial phyla in *I. scapularis* ticks from Texas and Massachusetts, USA. **Figure S3.** Bacterial richness (ACE and Chao1 estimators) in *I. scapularis* ticks. **Figure S4.** PCoA plot of weighted UniFrac distance metrics in male and female *I. scapularis* ticks collected from Texas and Massachusetts, USA. **Figure S5.** Unweighted PCoA plot of *Borrelia*-positive and *Borrelia-*negative *I. scapularis* males and females collected from Massachusetts, USA. **Figure S6.** Comparison of the bacterial microbiomes in colony-reared and field-collected *I. scapularis* ticks. (DOCX 1633 kb)

## Data Availability

All raw sequence data generated and/or analysed during this study are available in the National Center for Biotechnology Information (NCBI) Sequence Read Archive (SRA) with the accession number SRP144771 (https://www.ncbi.nlm.nih.gov/sra/SRP144771) under the BioProject PRJNA464062.

## Abbreviations

ACE: abundance-based coverage estimator
CDC: Centers for Disease Control and Prevention
MA: Massachusetts
OTU: Operational taxonomic unit
PCoA: Principal coordinate analysis
TX: Texas
