# Supplementary material for: Bacterial microbiomes of Ixodes scapularis ticks collected from Massachusetts and Texas, USA
Source: BMC Microbiol. 2019 Jun 24;19:138. doi: 10.1186/s12866-019-1514-7 (PMC6591839; doi:10.1186/s12866-019-1514-7)
Supplement: Supplementary file 1 — Figure S1. Rarefaction curves of the number of OTUs observed in male and female I. scapularis. Figure S2. Relative abundance of bacterial phyla in I. scapularis ticks from Texas and Massachusetts, USA. Figure S3. Bacterial richness (ACE and Chao1 estimators) in I. scapularis ticks. Figure S4. PCoA plot of weighted UniFrac distance metrics in male and female I. scapularis ticks collected from Texas and Massachusetts, USA. Figure S5. Unweighted PCoA plot of Borrelia-positive and Borrelia-negative I. scapularis males and females collected from Massachusetts, USA. Figure S6. Comparison of the bacterial microbiomes in colony-reared and field-collected I. scapularis ticks. (DOCX 1633 kb) [file 12866_2019_1514_MOESM1_ESM.docx]

**Additional files**

Bacterial microbiomes of *Ixodes scapularis* ticks collected from Massachusetts and

Texas, USA

Santosh Thapa^1#^, Yan Zhang^1^ and Michael S. Allen^1*^

^1^Tick Borne Disease Research Laboratory, Department of Microbiology, Immunology and

Genetics, University of North Texas Health Science Center, Fort Worth, Texas, USA

^#^ Current address: Texas Children’s Microbiome Center, Department of Pathology, Texas Children's Hospital & Department of Pathology and Immunology, Baylor College of Medicine, Houston, Texas, USA

* Corresponding author: Michael S. Allen, michael.allen@unthsc.edu.

Additional file 1 legends:

Figure S1. Rarefaction curves of the number of OTUs observed in male and female *I. scapularis.* Figure S2. Relative abundance of bacterial phyla in *I. scapularis* ticks from Texas and

Massachusetts, USA.

Figure S3. Bacterial richness (ACE and Chao1 estimators) in *I. scapularis* ticks.

Figure S4. PCoA plot of weighted UniFrac distance metrics in male and female *I. scapularis* ticks collected from Texas and Massachusetts, USA.

Figure S5. Unweighted PCoA plot of *Borrelia*-positive and *Borrelia-*negative *I. scapularis* males and females collected from Massachusetts, USA.

Figure S6. Comparison of the bacterial microbiomes in colony-reared and field-collected *I. scapularis* ticks.


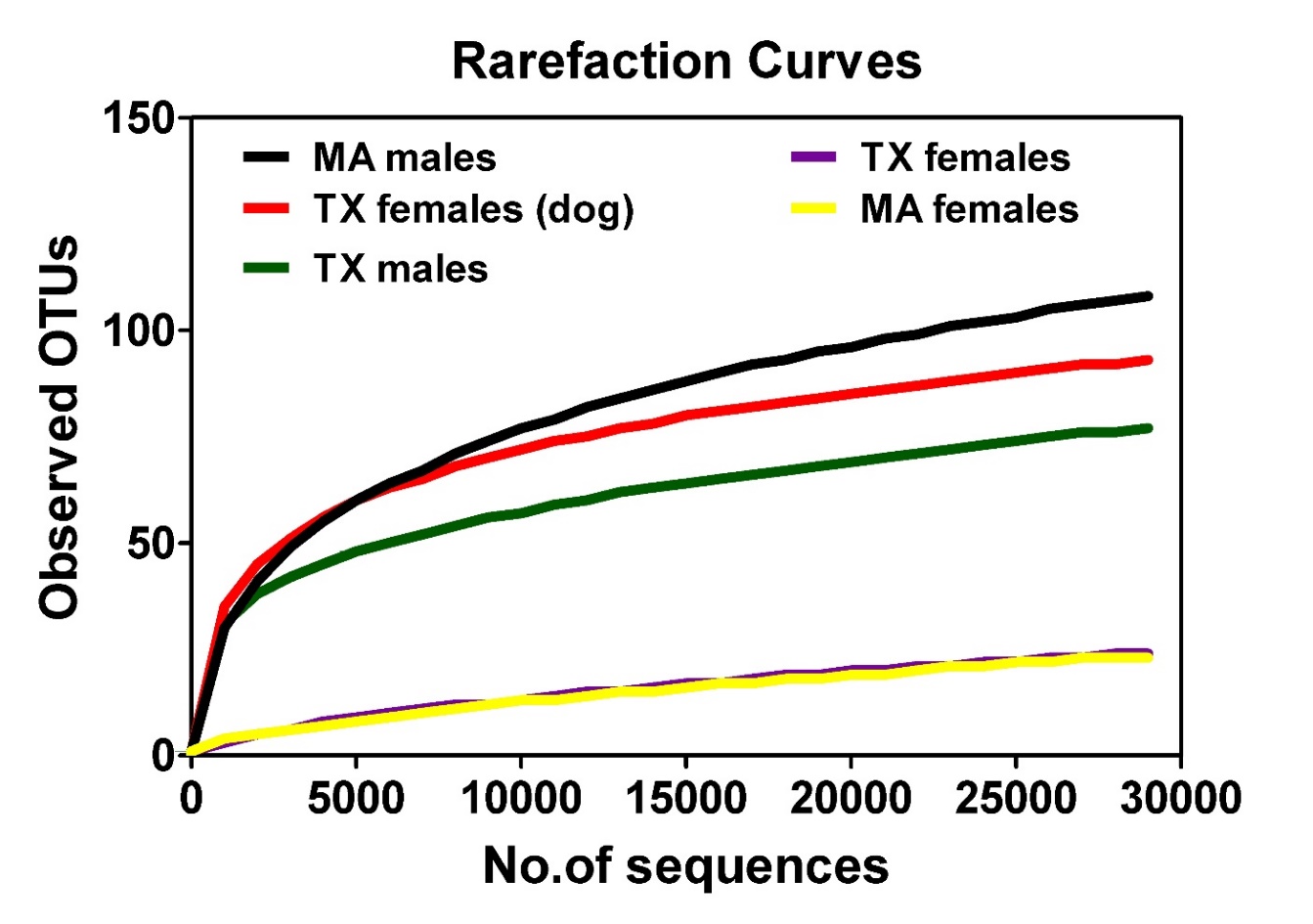


Figure S1. Rarefaction curves of the number of OTUs observed in male and female *I. scapularis*

**
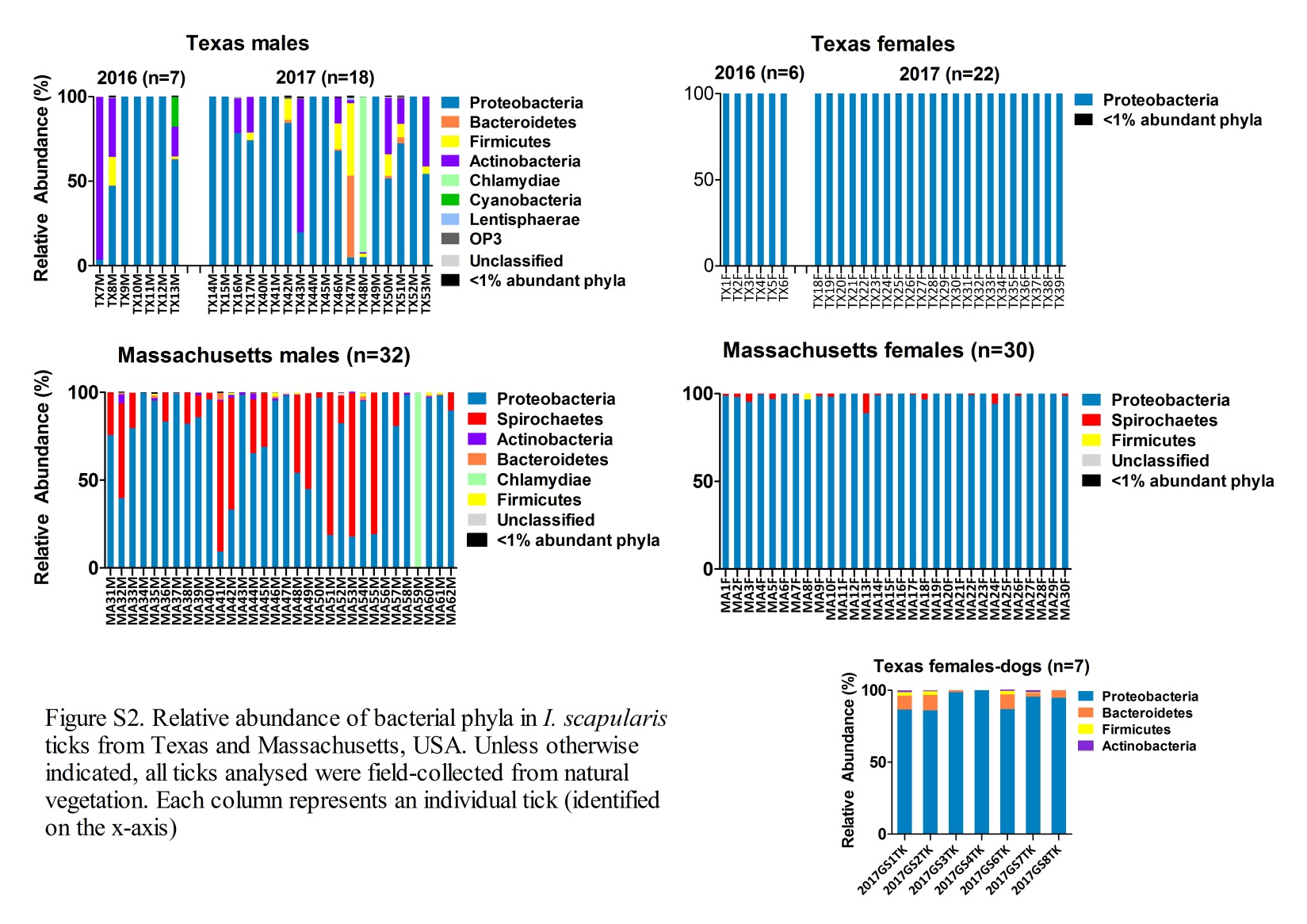
**

#

#


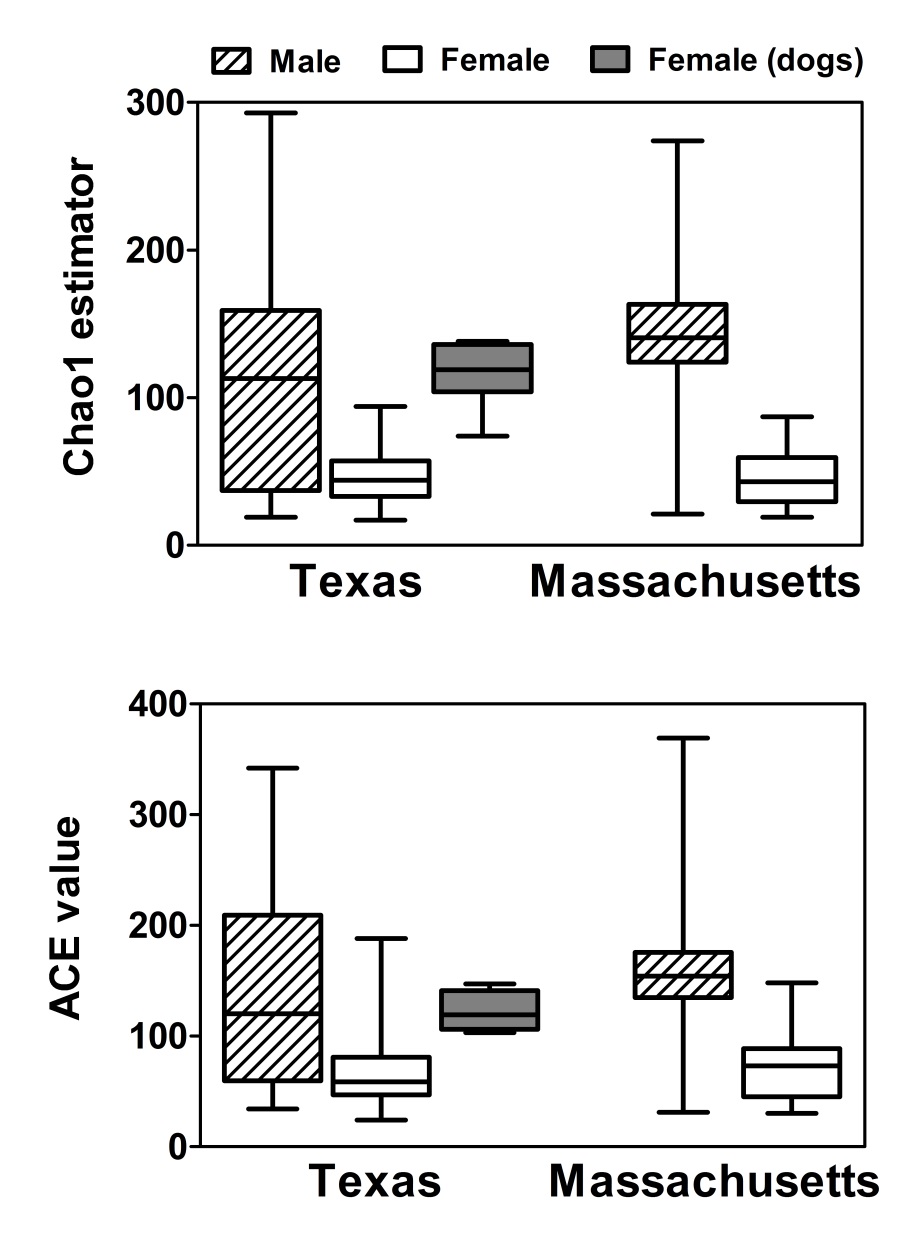


Figure S3. Bacterial richness (ACE and Chao1 estimator) in *I. scapularis* ticks. All ticks were collected from natural vegetation in Texas and Massachusetts, except female (dog) group where ticks were collected from dogs in North Texas. The ACE and Chao1 values for wild-caught male ticks (from both TX and MA) were significantly higher compared to that of the female ticks (Wilcoxon rank-sum test *p* < 0.05). Female ticks collected from Texas dogs had a significantly higher ACE and Chao1 indices in comparison to the female ticks collected from vegetation in both TX and MA (FDR corrected Wilcoxon rank-sum test *p<*0.0001 for all comparisons).


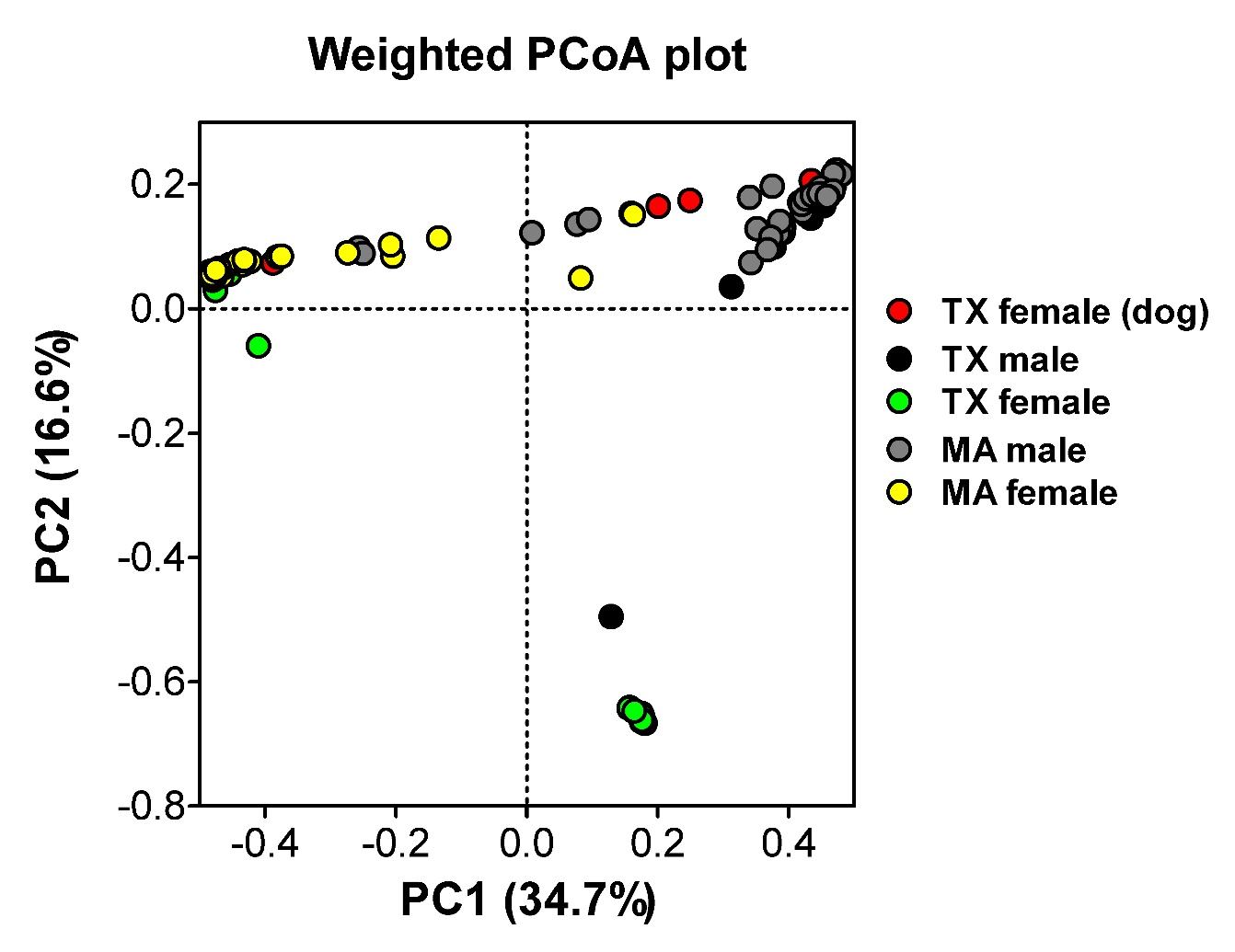


Figure S4. PCoA plot of weighted UniFrac distance metrics in male and female *I. scapularis* ticks collected from Texas and Massachusetts, USA.


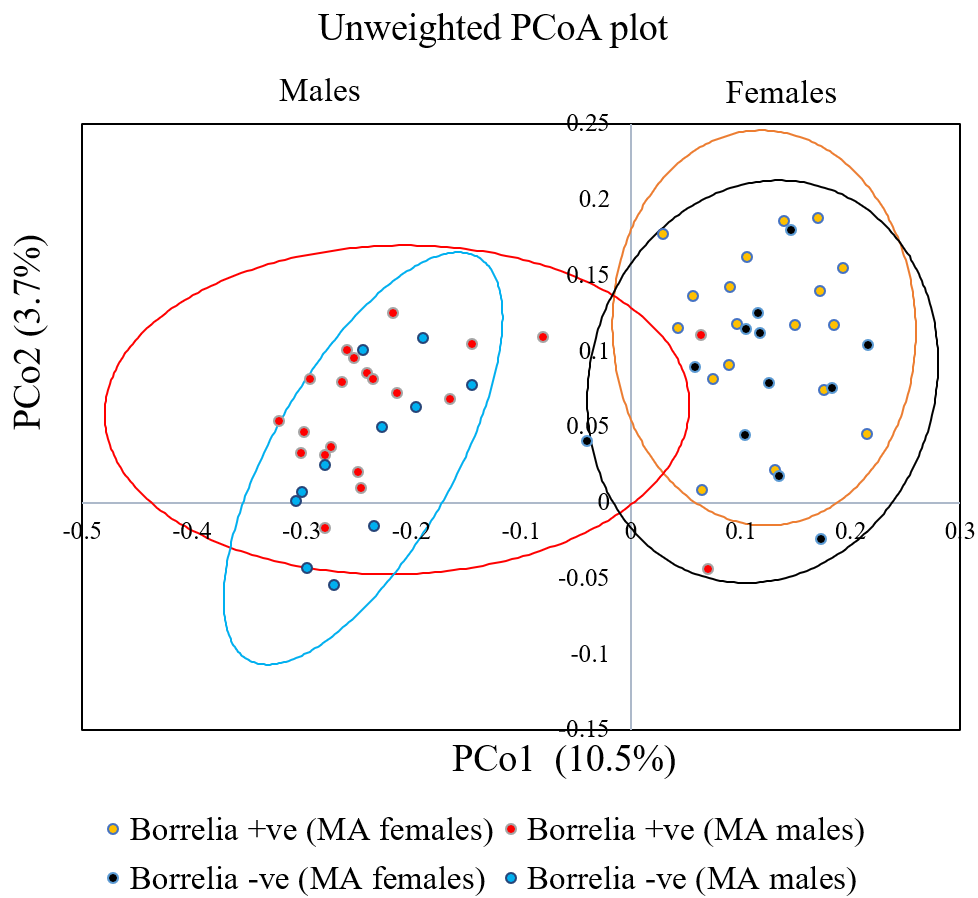


Figure S5. Unweighted PCoA plot of *Borrelia*-positive and *Borrelia-*negative *I. scapularis* males and females collected from Massachusetts, USA. The ellipses are drawn at 95% confidence intervals, where each dot represents the bacterial microbiome of an individual tick.


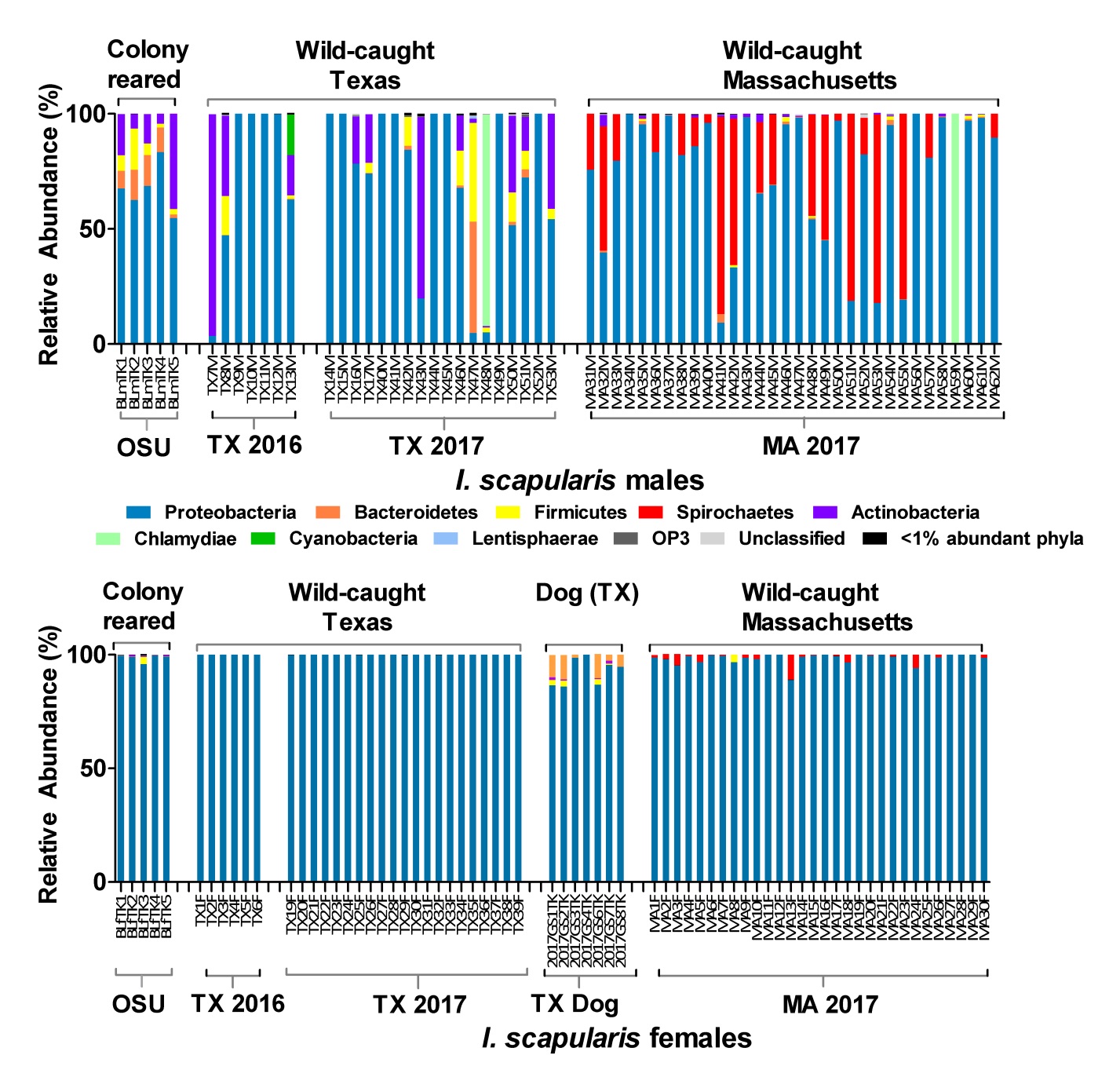


Figure S6. Comparison of the bacterial microbiomes in colony-reared and field-collected *I. scapularis* ticks. The ticks from natural vegetation in Texas were collected during 2016 and 2017 while the ticks from Massachusetts were collected in 2017. Five female ticks were collected from dogs in North Texas. OSU= colony-reared ticks purchased from the Oklahoma State University.
